# Identification and localization of bioactive naphthoquinones in the roots and rhizosphere of Paterson’s curse (*Echium plantagineum*), a noxious invader

**DOI:** 10.1093/jxb/erw182

**Published:** 2016-05-18

**Authors:** Xiaocheng Zhu, Dominik Skoneczny, Jeffrey D. Weidenhamer, James M. Mwendwa, Paul A. Weston, Geoff M. Gurr, Ragan M. Callaway, Leslie A. Weston

**Affiliations:** ^1^Graham Centre for Agricultural Innovation (Charles Sturt University and NSW Department of Primary Industries), School of Agricultural and Wine Sciences, Wagga Wagga NSW 2678Australia; ^2^Department of Chemistry, Geology and Physics, Ashland University, Ashland, OH 44805USA; ^3^Institute of Applied Ecology, Fujian Agriculture & Forestry University, Fuzhou 350002, China; ^4^Division of Biological Science, University of Montana, Missoula, MT 59812, USA

**Keywords:** Localization, periderm, plant secondary products, rhizosphere, shikonins, soil microprobes, SPRE, transport.

## Abstract

Bioactive plant secondary products are frequently the drivers of complex rhizosphere interactions, including those with other plants, herbivores and microbiota. These chemically diverse molecules typically accumulate in a highly regulated manner in specialized plant tissues and organelles. We studied the production and localization of bioactive naphthoquinones (NQs) in the roots of *Echium plantagineum*, an invasive endemic weed in Australia. Roots of *E. plantagineum* produced red-coloured NQs in the periderm of primary and secondary roots, while seedling root hairs exuded NQs in copious quantities. Confocal imaging and microspectrofluorimetry confirmed that bioactive NQs were deposited in the outer layer of periderm cells in mature roots, resulting in red colouration. Intracellular examination revealed that periderm cells contained numerous small red vesicles for storage and intracellular transport of shikonins, followed by subsequent extracellular deposition. Periderm and root hair extracts of field- and phytotron-grown plants were analysed by UHPLC/Q-ToF MS (ultra high pressure liquid chromatography coupled to quadrupole time of flight mass spectrometry) and contained more than nine individual NQs, with dimethylacrylshikonin, and phytotoxic shikonin, deoxyshikonin and acetylshikonin predominating. In seedlings, shikonins were first found 48h following germination in the root-hypocotyl junction, as well as in root hair exudates. In contrast, the root cortices of both seedling and mature root tissues were devoid of NQs. SPRE (solid phase root zone extraction) microprobes strategically placed in soil surrounding living *E. plantagineum* plants successfully extracted significant levels of bioactive shikonins from living roots, rhizosphere and bulk soil surrounding roots. These findings suggest important roles for accumulation of shikonins in the root periderm and subsequent rhizodeposition in plant defence, interference, and invasion success.

## Highlight

Napthoquinones, antimicrobial and phytotoxic metabolites that are involved in plant defence, are produced and released into the rhizosphere by root hairs and root periderm tissue of *Echium plantagineum.*

## Introduction

### Plant secondary products (PSPs) and root interactions

Although our knowledge of root structure and function has improved in recent years, the complex activities and interactions of roots in the soil rhizosphere and at the soil-root interface are often poorly described, and this is particularly true for invasive weeds. It has become increasingly evident that both root exudation and rhizodeposition in plants are responsive to biotic and abiotic stressors and are clearly important for the protection of sessile terrestrial plants, particularly in the immediate area surrounding living roots, otherwise known as the rhizosphere (Bertin *et al.*, 2003; Badri *et al.*, 2009; Watt and Weston 2009; Weston *et al.*, 2012a). Over time, living roots accumulate and release bioactive plant secondary products (PSPs) from various root tissues, creating both physical and chemical barriers against penetration by plant pathogens, microbes and herbivores (Hutzler *et al.*, 1998; Weston and Duke 2003; Callaway *et al.*, 2008). Protective mechanisms used by living plants have recently been explored in some detail for selected crop or medicinal plants and their resulting cell suspension cultures (Weston *et al.*, 2012a, 2013a; Yazaki, 2005; Ozgen *et al.*, 2011; Wink, 2015).

The accumulation of PSPs in specialized tissues and organs in living roots and their potential role in rhizosphere defence have been documented for several crop and medicinal species. For example, the distribution of bioactive glucosinolates in the periderm of canola roots with respect to their role as soil fumigants and plant protectants was studied by McCully *et al.* (2008). In 2001, Czarnota *et al.* first described the role and mode of action of phytotoxic sorgoleone and related long chain hydroquinones produced by living sorghum root hairs as plant growth inhibitors, and also described localization and release of sorgoleone by living root hairs (Czarnota et al., 2003; Weston *et al.*, 2012a, 2013a). The saponin avenicin was identified in oat root tips by Osbourn in 1996 and its activity and localization as an antifungal agent and plant protectant in roots was later described by Morrissey and Osbourn (1999). The role of flavonoids in legume roots, legume nodulation and rhizobium signalling processes has also been well documented and more recently flavonoids have been shown to mediate allelopathic interactions in the plant rhizosphere (Carlsen and Fomsgaard, 2008; Mathesius and Watt, 2011; Weston and Mathesius, 2013).

Phenolic constituents such as flavonoids are also important in structural plant protection (Harborne, 1999). Phenylpropanoid and flavonoid molecules accumulate in both guard cells and epidermal cells, on outer layers of organs, in waxes, or may be covalently linked to plant cell walls (Schnabl *et al.*, 1989; Hrazdina and Jensen, 1992; Hutzler *et al.*, 1998). Numerous studies have indicated a high degree of compartmentalization of phenylpropanoids and flavonoids, and enzymes responsible for their production. However, most root-produced compounds of interest remain to be evaluated in terms of their accumulation over time in various plant tissues and organs. As Hutzler *et al.* (1998) suggest, a basic understanding of the ecological function of phenolic compounds requires a simultaneous understanding of the structure of the compounds of interest, their biosynthetic pathways and regulation and also their tissue localization. Recent developments in microscopy techniques including confocal laser scanning microscopy (CLSM) have provided opportunities to study localization of PSPs, including phenolics, more precisely than the use of conventional brightfield and fluorescence microscopy. In particular, CLSM allows for identification of compounds of interest by studying specific fluorescence characteristics, including emission and absorption (Sheppard, 1993; Hutzler *et al.*, 1998).

Currently, detailed information is very limited on the anatomy of invasive plant roots, the role of associated secondary products in plant protection, interference with plant growth and subsequent plant invasion. Although Callaway (2000), Callaway and Aschehoug (2000) and Callaway *et al.* (2008) outline the possible role of PSPs released by root exudates in plant invasion, the specific study of their localization in plant roots and the release mechanisms of allelochemicals by invasive plants have rarely been documented. However, PSPs are known to be important in influencing rhizosphere interactions among noxious weedy species, including those with neighbouring native plants as well as microbial associations (Hierro and Callaway 2003; Stinson *et al.*, 2006; Callaway *et al.*, 2008; Inderjit *et al.*, 2011).

Recent breakthroughs in the study of the plant rhizosphere have reported on root-associated microbiomes (Edwards *et al.*, 2015) and the identification of novel microbial metabolites with activity as potent antibiotics or quorum sensing agents (Weston and Mathesius, 2013). However, fewer studies have actually documented the release of plant- or microbially-produced metabolites or ‘novel weapons’ influencing plant invasion success, particularly with respect to their localization in roots, the rhizosphere or in bulk soil (Weidenhamer and Callaway, 2010; Inderjit *et al.*, 2011). This is most certainly due to the difficulty in identification of trace quantities of PSPs in roots and the soil rhizosphere, and the fact that the soil rhizosphere interface can be an incredibly complex and dynamic matrix that is difficult to survey or potentially extract. The recent development of techniques that allow for dynamic profiling of non-polar to moderately polar root-produced PSPs in the soil rhizosphere with silicone tubing and solid phase root zone extraction has facilitated more precise and direct profiling of certain moderately polar to non-polar bioactive molecules released by living plant roots, as in the case of *Sorghum bicolor* and *Tagetes erecta.* These techniques have also allowed for consideration of spatial mapping of PSPs and their deposition within the living plant rhizosphere (Mohney *et al.*, 2009; Weidenhamer *et al.*, 2009).

### Paterson’s curse (*Echium plantagineum*), an important invader in Australia

In Australia*, Echium plantagineum* L., commonly known as Paterson’s curse or salvation Jane, is a noxious weed infesting more than 30M ha of crop and rangeland (Piggin, 1982; Grigulis *et al.*, 2001). It is native to the Iberian Peninsula, specifically the eastern regions of Spain and Portugal, and is now naturalized across much of southern Australia, parts of the Mediterranean, the USA and South Africa (Piggin, 1982; Weston et al., 2012b, 2013b). Introduced in the mid 1800s to Australia, the initial distribution of Paterson’s curse is likely associated with the frequent importation of Merino sheep, or as an accidental contaminant of pasture seed and hay (Zhu *et al.*, 2014). In recent years its range has increased (Weston *et al.*, 2013b) and it often dominates plant communities in poor, drought-prone soils to the extent that it costs the wool and meat industries more than A$250M per year in losses due to reduced livestock productivity (NRM South and the Southern Tasmanian Councils Authority, 2015).

### Bioactive plant secondary products in *Echium plantagineum*

*E. plantagineum* produces significant quantities of several important PSPs including pyrrolizidine alkaloids in its leaves, stems, flowers and seeds that can cause liver, kidney and lung damage in mammals, eventually poisoning horses, sheep and cattle that have consumed sufficient quantities of foliage (Peterson and Jago, 1984; Colegate *et al.*, 2005; Quinn *et al.*, 2014; Skoneczny *et al.*, 2015). However, in addition to pyrrolizidine alkaloids, *E. plantagineum* also produces unusual bright red-coloured naphthoquinones (NQs) in its roots. Analysis of many field-collected roots of *E. plantagineum* by the authors revealed that the outer layers of root tissue in the primary taproot or smaller fibrous secondary roots are very often pink or red due to the production of a mixture of bioactive, brightly coloured NQs known as shikonins (Weston et al., 2012a, b). NQ content typically increases in roots of summer-collected plants in comparison to those sampled in winter or spring. In addition, geographically distinct populations of *E. plantagineum* collected from warm, dry roadside locations across New South Wales (NSW) at low elevations produced significantly (3–5-fold) higher concentrations of NQs than plants collected from similar sites with cooler average temperatures or higher elevations (Weston *et al.*, 2013b).

Although the production of shikonins is unusual in higher plants, the roots of numerous members of the Boraginaceae, including species of *Alkanna*, *Arnebia* and *Lithospermum* as well as *Echium,* contain numerous shikonins (Papageorgiou *et al.*, 1999; Sommer *et al.*, 1999; Boehm *et al.* 2000). In the medicinal literature, these compounds are referred to as shikonins, alkannins or naphthazarins and have been the subject of numerous studies due to their activity as antioxidants, antihelminthics and purgatives, and as aids in wound-healing (Papageorgiou *et al.*, 1999; Assimopoulou *et al.*, 2006; Hu *et al.*, 2006; Albreht *et al.*, 2009). They have also been reported as curatives for prostate cancer due to their ability to induce cell apoptosis (Gara *et al.*, 2015). Shikonins from the Boraginaceae specifically exhibit potent antibiotic activity against certain gram-negative bacteria (Papageorgiou *et al.*, 1999). Strong antagonistic effects of shikonin and other naphthoquinones on other plants, insects, fungi and bacteria have also been observed; activity is likely associated with the potent inhibition of electron transport processes by NQs, particularly upon respiration, but cell division or other cellular processes may also be impacted (Binder *et al.*, 1989; Brigham *et al.*, 1999; Babula *et al.*, 2009; Weston et al., 2012a, b). Both purified NQs, including shikonin and acetylshikonin and root extracts of field-grown Australian *E. plantagineum* showed potent activity on plant growth, in contrast to similar concentrations of extracts from Spanish plants (Garcia Duran *et al.*, 2014, 2015).

In order to gain a more fundamental understanding of the ecological role of NQs in *E. plantagineum* root tissues and also the rhizosphere of this invasive plant, we employed both confocal and light microscopic imaging techniques to perform anatomical investigations of living roots of this weedy invader, along with UHPLC/Q-ToF MS (ultra high pressure liquid chromatography coupled to quadrupole time of flight mass spectrometry) to perform metabolic profiling of root, root hair and soil extracts. We also utilized SPRE (solid phase root zone extraction) microprobes in the soil rhizosphere to profile PSPs of interest in the rhizosphere and bulk soil surrounding living plant roots to further define the role of PSPs as potential drivers of plant/plant and plant/organismal interactions in the rhizosphere.

## Materials and methods

### NQ localization experiments in living plant tissue using confocal and light microscopy

*Echium plantagineum* root tissues were collected from densely populated local field stands in Wagga Wagga, NSW, Australia (−35.0586°N, 147.3507°E) in 2014. At least five mature flowering specimens were collected for microscopic evaluation of NQs at various intervals from August to October 2014. On all occasions following collection, plants were placed in wet paper towelling to prevent dehydration and maintained at a temperature of 4 °C until evaluation under a microscope, which was typically performed within 1h of collection. *E. plantagineum* seed was also collected from the same field location in Wagga Wagga, NSW, in 2013 and used to generate seedlings for time-course experimentation of NQ production under controlled environmental conditions. Seeds were germinated in June 2014 on Whatman No. 1 filter paper moistened with 5ml sterile deionized water in sterile 9-cm plastic Petri dishes containing 20 seeds per dish, with three replicates for each harvest time interval (*n*=60 seedlings). Harvest times were 12, 24, 48, 72, 96, 128 and 144h after experimental initiation. During incubation, dishes were sealed with parafilm and placed in a lighted incubator at 25/18 °C day/night temperatures with a 12-h photoperiod; microscopic evaluation was performed immediately following each harvest.

Mature roots and seedling root and hypocotyl tissues were hand sectioned for examination using confocal microscopy (Nikon A1 Confocal TiE inverted microscope, excitation 488nm, emission 570–620nm for NQ/shikonin evaluation). In most cases, sections were directly examined without staining, however calcofluor white (1% aqueous solution) (Sigma Aldrich, Australia) was used on a few occasions for more pronounced staining of the cell wall. The fluorescence from calcofluor white was detected with an excitation of 405nm and emission from 425 to 475nm. The same confocal microscope was used for both hyperspectral analyses and imaging to compare *E. plantagineum* fresh root periderm spectra with those generated with an ethanolic solution of pure shikonin (Sigma Aldrich, Australia) at a concentration of 1mg ml^−1^. For spectral analyses, images were scanned sequentially with excitation wavelength of 405 and 488nm, with a wavelength interval of 6nm and emission range from 405–550 and 550–740nm, respectively. The two spectral scans were then automatically combined by Nikon confocal NIS ver 4.10 to create the emission spectrum presented. For seedlings generated during the time course experiment, stereoscopic light microscopy using both a Nikon SMZ25 and a Leica M205FA was performed at each time interval with numerous sampled seedlings.

### Microprobe experiments to assess NQs in rhizosphere and rhizosphere soil extraction

Solid phase root zone extraction (SPRE) microprobes (5cm) were prepared as per Weidenhamer *et al.* (2009) using silicone polydimethylsiloxane (PDMS) Silastic tubing (Fisher Scientific, USA), supported internally with fine stainless steel wire (22 gauge), as a probe for entrapment of nonpolar soil rhizosphere PSPs such as NQs. To fully evaluate whether microprobes could be used to entrap and collect NQs to intensify concentrations of these compounds in a rhizosphere setting, eight sets of three microprobes each were placed in contact with live roots collected from mature greenhouse-grown plants. Roots were sectioned into 5cm long pieces and placed in contact with microprobes for 1 or 12h in each of eight sterile Petri dishes containing moistened filter paper. After treatment, unexposed microprobes (those not subjected to root exposure and therefore serving as a negative control) and root-exposed microprobes were photographed. All unexposed controls and those microprobes exposed to roots for 12h were then extracted in HPLC grade 100% ethanol (VWR Chemicals, Australia) for 10min, followed by evaporation of extracts under a stream of N_2_ gas for approximately 20min. The resulting dried extract was weighed and resuspended in ethanol, filtered through a 22 μm Millex syringe filter and subjected to UHPLC/Q-ToF MS analysis as described below.

Greenhouse-grown plants were propagated for 16 weeks in a soil mix containing 6:4 peat potting mix:sand (Scotts Co., Melbourne, Australia.). Seedlings were pre-germinated by imbibing in sterile water for 1 week using field-collected seed as described above in NSW, Australia; after 7 d, seedlings were transplanted into 1.5-l pots and were maintained in the glasshouse at 25/18 °C day/night temperatures at ~55% relative humidity. Plants were watered every other day by subirrigation and fertilized once per fortnight using a commercial liquid fertilizer (N:P:K=23:3.95:14, Aquasol Soluble Fertilizer, Australia). At 14 weeks of age, as plants began to flower, plants were sampled for rhizosphere-produced NQs using 5cm microprobes, as described above. Eight probes were placed equidistantly into each of six plant pots and were fully inserted into the soil surrounding the living plant, ~8cm from the centre of the plant rosette. The probes were placed equidistantly around the plant at a distance of ~5cm from the taproot, and were not in contact with the foliage of the plant.

In addition, soil media was collected separately from the rhizosphere (soil not adhering to plant roots but located around living plant root system) of each pot, thoroughly filtered to remove any small residual root pieces using a fine wire mesh screen (<1mm mesh holes), air-dried and extracted in 100% ethanol, filtered using a 22 μm Millex filter and subjected to analysis using an ion trap mass spectrometer for NQ detection.

### Chemical extraction of field-collected root samples

Specimens of *E. plantagineum* were collected in the field from 21 locations across NSW and Australian Capital Territory, Australia, in 2013 and 2014. At each collection site, GPS coordinates were recorded and five or more intact root specimens were collected from mature, flowering plants. Roots were carefully collected from field sites using a mattock to remove the majority of the root system without excessive damage to the taproot and main secondary roots. Excess soil was removed and roots were placed in moist paper towelling and stored at 4 °C for 24h prior to extraction. Root periderm extracts were prepared by thinly peeling the coloured outer periderm layer only from taproots or primary roots using a sharp scalpel blade. To minimize the impact of plant-to-plant variation of extracts to be subjected to metabolic profiling, composite samples were prepared from 5–6 individual plant roots collected at each of 21 locations using ~0.2g of fresh periderm per plant to generate 1g total fresh weight of periderm tissue. Periderm peels (1g) were then extracted in 10ml of 100% HPLC grade ethanol (VWR Chemicals, Australia) for 14h in the dark, at room temperature, after placement on a slow orbital shaker at 120rpm. Following extraction, samples were filtered using a 22 μm Millex syringe filter, and 1ml of each extract was transferred into individual HPLC vials with duplicate samples available for replicated UHPLC/Q-ToF MS profiling (Weston *et al.*, 2015). A similar protocol was used to extract fresh root periderm tissues of seedlings grown in controlled environments; in this case up to 0.25g of fresh tissue was extracted from 10 seedlings in ethanol to provide sufficient sample for further UHPLC/MS analysis.

### UHPLC/Q-ToF MS analyses

Metabolic profiling of key NQs in root periderm extracts was performed using an Agilent 1290 Infinity UHPLC system equipped with quaternary pump, degasser, temperature controlled column and cooled autosampler coupled to an Agilent 6530 Quadrupole Time-of-Flight (QToF) mass spectrometer with Dual Agilent Jet Stream Electrospray Ionisation Source (Dual AJS ESI) (Agilent Technologies, Mulgarve, Australia) (Weston *et al.*, 2015). Separation was achieved using a C_18_ Poroshell column (2.1×100mm, 2.7µm) at 25 °C equipped with an SB-C_8_ guard column (2.1×12.5mm, 5µm) (Agilent, Santa Clara, CA, USA) and a flow rate of 0.5ml min^−1^. The column was equilibrated for 30min prior to analysis. Solvents used for extraction and UHPLC/MS were HPLC grade. Acetonitrile was obtained from Hipersolv (Tingalpa, Australia), formic acid (>99% purity) from Sigma (Castle Hill, Australia) and LC-MS water from Merck (Darmstadt, Germany). Separation of NQs was achieved using a gradient of mobile phase A (water+0.1% formic acid) and mobile phase B (95% acetonitrile+0.1% formic acid), starting with 50% B for 1min and reaching 100% B over 7min, continuing at 100% B until 10.50min, returning to 50% B over 0.1min and held at 50% B from 10.60–17.00min. (Skoneczny *et al.*, 2014). The QToF was run and calibrated in negative ion mode, with nebulizer gas at 35 psig, capillary voltage at 3500V and fragmentor voltage at 135V. Nitrogen was used as drying gas at 250 °C at a flow of 9 l min^−1^. Data were collected in negative ion mode using an extended dynamic range (2 GHz). Additional LC/MS-MS experimentation was performed for selected molecules of interest (Huang *et al.*, 2010) using analytical standards of acetylshikonin (MW 330.1103; RT 5.75 min±0.7min), deoxyshikonin (MW 272.1048; RT 6.75±0.7min), dimethylacrylshikonin (MW 370.1416; RT 7.7 min±0.7min) purchased from ChemFaces (Wuhan, China) and shikonin (MW 288.0997; RT 3.51 min±0.7min) obtained from Biomol (Hamburg, Germany). All data were analysed using MassHunter software (ver. B.07, Agilent, Santa Clara, CA, USA).

### Q-Trap HPLC/MS analyses

Further analyses of NQs in soil and soil microprobe extracts at trace concentrations were performed using Agilent 1200 series HPLC coupled to an ABSciex 3200 Q-Trap mass spectrometer (AB Sciex, Foster City, CA, USA) and using analytical standards (as described in the previous section) and HPLC grade solvents including methanol from Rathburn (Walkerburn, Scotland) and formic acid from Merck (Darmstadt, Germany). Separation was achieved using Kinetex XB-C_18_ 2.1×100mm, 2.6 μm, 100 Å (Phenomenex, Macclesfield, UK) column with a gradient of solvent A (water, 0.02% formic acid) and solvent B (100% methanol, 0.02% formic acid). The gradient was initiated with 40% B for 1min and reached 98% B over 9min, continued at 100% B until 13.0min and returned to 40% B over 0.1min and held at 40% B from 13.1–18.50min. Optimization of the MRM transitions for purified standards was performed using standards formulated at 5ppm (in 50:50 methanol:water) that were injected into the MS/MS interface using syringe infusion at 10 µl min^−1^. To determine specific precursor and product ions for each standard, MS/MS was performed, and after optimization shikonins were evaluated in negative ion mode (Table 1). Data were analysed using Analyst 1.5 software (AB Sciex, Foster City, CA, USA).

**Table 1.** Optimized values of compound dependent parameters for purified analytical standards of shikonin, deoxyshikonin, acetylshikonin and dimethylacrylshikonin obtained using ABSciex 3200 QTrap mass spectrometer (AB Sciex, Foster City, CA, USA)

Compound name	Q1 (precursor ion)	Q3 (daughter ion)	Declustering potential	Entrance potential	Cell entrance potential	Cell exit potential
Deoxyshikonin	271.821	203.1	−40	−5	−28	−4
Shikonin	286.901	217.9	−25	−1	−18	−4
Acetylshikonin	328.923	269.1	−25	−5	−26	−4
Dimethylacryl shikonin	368.782	269.0	−20	−5	−20	−4

### Statistical analysis

Abundance of deoxyshikonin, shikonin, acetylshikonin and dimethylacrylshikonin was analysed in 21 samples, representing 21 populations. Analysis of variance was performed on log transformed data in IBM SPSS statistics software (IBM Corp., NY, USA). Homogeneity of variances was assessed using Levene’s test prior to further analysis. Tukey HSD was used as a post hoc test to evaluate differences among metabolite levels averaged over populations.

## Results and discussion

**Fig. 1.**
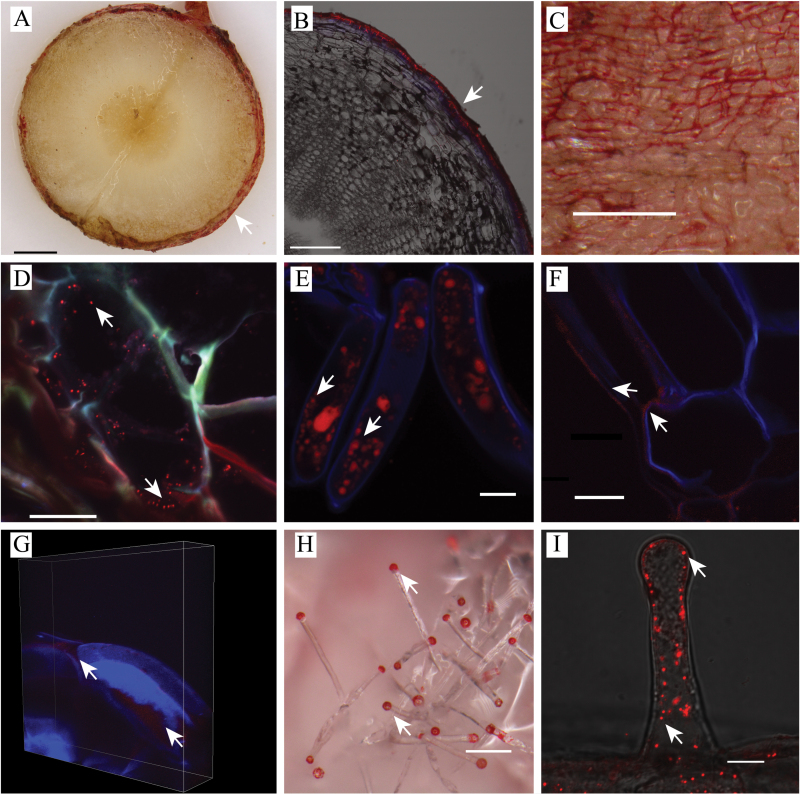
Localization of shikonins in *E. plantagineum*. Under bright field and confocal miroscopy shikonins were bright red in colour. (A) Bright field image of mature taproot cross section showing the red-coloured periderm cells (arrow). Bar, 1mm. (B) Sequential scanning confocal image of mature secondary root cross section showing autofluorescence of the periderm tissue corresponding to shikonin localization (arrow). Bar, 250 µm. (C) Bright field image of mature taproot surface showing shikonin deposition in mature root. Bar, 250 µm. (D) Confocal image of a typical periderm cell of mature plant containing numerous small vesicles (arrows). Bar, 20 µm. (E) Confocal image of selected intact root epidermal cells from a 6-day-old seedling, showing numerous vesicles (arrows). Tissue was stained with calcofluor white (blue). Bar, 10 µm. (F) Sequential scanning confocal image of outer periderm cells of a mature plant, showing shikonins localized in extracellular areas (arrows). Tissue was stained with calcofluor white (blue). Bar, 10 µm. (G) Sequential scanning 3-D image of outer periderm cells of a mature plant, showing shikonins localized in extracellular areas (arrows). Tissue was stained with calcofluor white (blue). Width, 127.15 µm; height, 127.15 µm; depth, 28.50 µm. (H) Bright field image of root hair of 3-day-old seedling, showing root hair exudation of shikonins (arrows). Bar, 200 µm. (I) Confocal image of root hair in (H), showing numerous vesicles throughout the root hair (arrows). Bar, 10 µm.

**Fig. 2.**
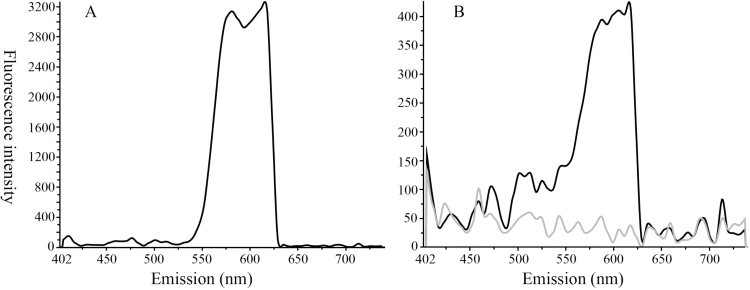
Spectral imaging of (A) pure shikonin in ethanol (concentration of 1mg ml^−1^ in ethanol) in comparison to (B) (black line) outer periderm cells of mature *E. plantagineum* root containing shikonins using multiple spectral scans at excitation wavelengths of 405 and 488nm, and (B) (grey line) control spectral scan of root cortex which is devoid of shikonins. Respective fluorescence emission (peak maxima at ~560 and 620nm) is very similar for an analytical standard of shikonin and hyperspectral scan of root periderm *in situ*.

In both field and glasshouse raised *E. plantagineum* roots, shikonins were clearly identified in the outer one to two cell layers of newly formed periderm in mature and seedling taproots as well as secondary roots (Fig. 1 A–C). The periderm is defined as the protective outer cortical layer present in many roots and stems of dicots; this layer can also contain secondary plant products likely involved in plant protection (McCully *et al.*, 2008). Shikonin presence was denoted by red colouration of the outer periderm and/or autofluorescence at 488nm, as reported by Papageorgiou *et al.* (1999). Similar to localization of glucosinolates in canola periderm (McCully *et al.*, 2008), shikonins were found only in outer periderm tissues and not interior root cortical tissues as observed by microscopy and evaluation by UHPLC/Q-ToF MS (Table 1). Shikonins are both UV absorptive and autofluorescent, and can be highly coloured, ranging from pink to red or purple, depending on concentration and pH. Confocal microspectrofluorimetry was therefore used to confirm the presence of shikonins *in situ* in mature periderm tissue of field-collected plants by scanning over an emission spectrum ranging from 405 to 740nm in comparison to a known standard of shikonin. Nearly identical spectroscopic results were obtained from both scans, suggesting that compounds present in mature periderm tissue of *E. plantagineum* are identical or closely related to the bioactive naphthoquinone shikonin (>98% pure standard of molecular weight=288.3) both in their unique colouration and their autofluorescence (Fig. 2). Upon closer examination of mature periderm cells under greater magnification, we clearly observed the presence of numerous small red-coloured vesicles in the interior of the cell (Fig. 1D, E), suggesting that incorporation into vesicles is a means of transport of PSPs such as shikonins in the cell, and likely also serves to protect intracellular organelles and processes against autotoxicity associated with the presence of naphthoquinones such as shikonins, which exhibit potent inhibition of respiration and electron transport processes (Babula *et al.*, 2009; Weston *et al.*, 2012a, 2013a).

Confocal analysis also revealed that shikonins accumulated in large quantities extracellularly by deposition outside of the cell in extracellular spaces over time or possibly in association with plant cell walls through covalent bonds (Fig. 1F, G). Phenylpropanoid and flavonoid molecules also accumulate in outer layers of plant organs, in waxes, or are even covalently linked to plant cell walls (Schnabl *et al.*, 1989; Hrazdina and Jensen, 1992; Hutzler *et al.*, 1998). It is possible that shikonins may play a role in structural integrity as well as exhibiting both phytotoxic and antimicrobial activity in the plant periderm and rhizosphere (Brigham *et al.*, 1999; Garcia Duran *et al.*, 2014). The extracellular deposition of shikonins suggests that they may play a role in structural integrity of the periderm layer over time and/or these PSPs are exported to prevent autotoxic build-up in the dynamic intracellular environment of a specialized periderm cell. Further labelling studies would help to determine if NQs are incorporated into the cell walls of periderm tissues or are just deposited in extracellular spaces.

Field and glass-house grown plant examination revealed that shikonins were also released by direct exudation in droplets which accumulated at the tips of living root hairs. We observed this phenomenon not only in mature plant root hairs but also in seedlings grown in Petri dishes within 48h of germination and radicle elongation. It is evident that considerable exudation occurs in seedlings as noted by the copious quantities of red-coloured exudates observed accumulating at the tips of living root hairs (Fig. 1H). Confocal analyses indicated the presence of numerous small vesicles present in living root hair cells (Fig. 1I), which are likely associated with shikonin intracellular transport and exudation. Root hair exudation in *E. plantagineum* is strikingly similar to that observed in living sorghum roots, which release large quantities of sorgoleone accumulating in vesicles at the tips of living root hairs. In the case of non-polar long chain hydroquinones such as sorgoleone, exudation is reported to occur by direct extrusion through spaces or pores in the plasmalemma (Weston *et al.*, 2012b, 2013a). Further experimentation is required to determine if protein transporters are associated with the movement and deposition of moderately polar to non-polar shikonins to extracellular spaces, and to quantify the relative availability and abundance of these compounds on the surface of mature taproots and secondary roots which may not possess the large numbers of living root hairs found on seedlings of *E. plantagineum*. The living periderm is continually replaced in dicots, and over time significant rhizodeposition of shikonins may occur due to the continuous sloughing off and degradation of periderm tissue, as indicated for canola by McCully *et al.* (2008). This suggests that NQs could be continually replenished into the rhizosphere during the life cycle of a biennial such as *E. plantagineum*.

However, similar to sorgoleone exudation which increased in stressed plants, Brigham *et al.* (1999) noted that production of shikonins in root suspension cultures increased over time with exposure to stressors such as temperature and extracts containing fungal cell walls, which serve as elicitors of shikonin production. We also noted this correlation in field experiments which detected increased production of shikonins in plant roots that were collected from field sites experiencing greater temperature, lower elevation and likely drought stress (Weston *et al.*, 2013b).

**Fig. 3.**
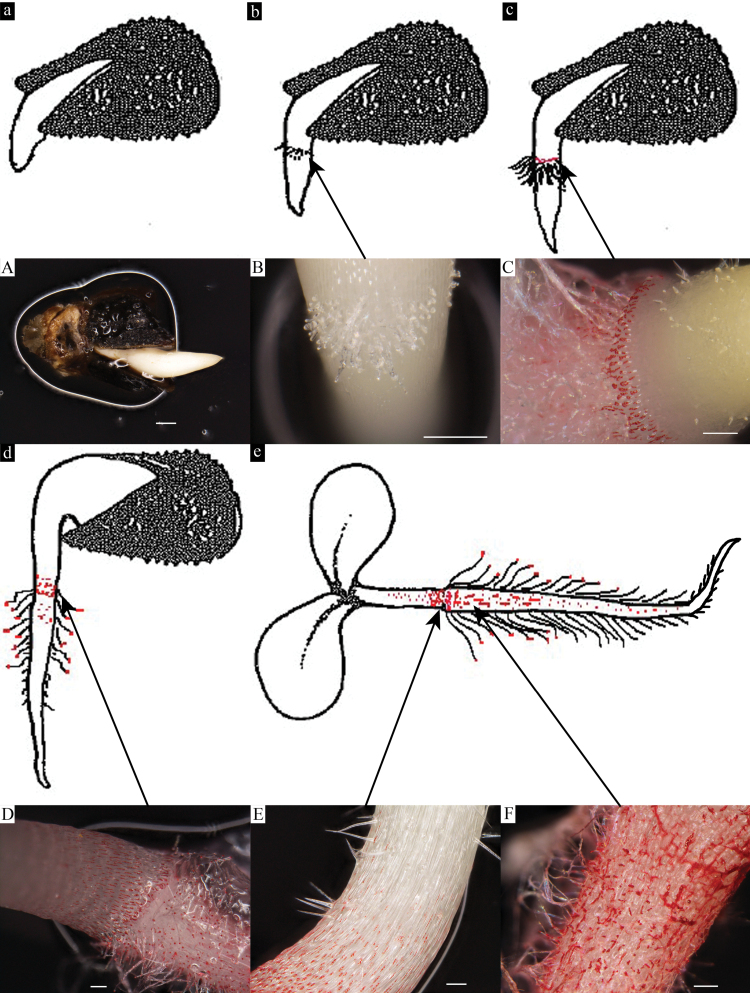
Time course experiment of *E. plantagineum* seedlings (a–e) at 24, 36, 48, 72 and 120h after germination. Note the location of shikonin production noted by red colouration as visualized on the radical and hypocotyl. Photos A–D correspond to seedlings at phenological stages a–d, respectively and photos E–F to radical and hypocotyl stages noted in panel e. (A) Seedling 24h after imbibition. (B) Seedling 36h after imbibition, showing newly emerging root hairs. (C) Seedling 48h after imbibition, showing shikonin localization in root primordial zone and numerous exuding root hairs. (D) Seedling 72h after imbibition, showing shikonin production in hypocotyl, radicle and root hairs. (E) Hypocotyl of seedling at 120h following imbibition, showing shikonin production in zone of differentiation between radicle and hypocotyl. (F) Radicle at 120h after imbibition, showing distinct mature root hairs producing shikonins and extensive shikonin accumulation in developing periderm. Bars, 500 µm (A); 200 µm (B–F).

**Fig. 4.**
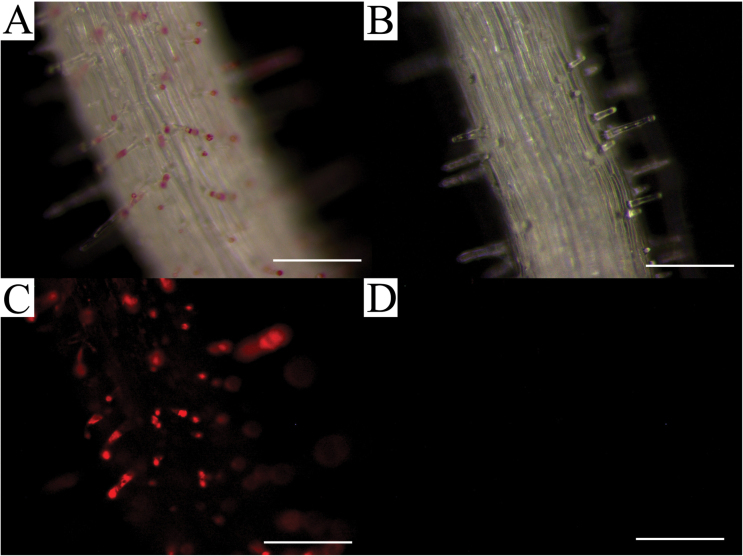
Radicle 72h after imbibition. (A) Bright field image of more mature (upper) radicle showing root hair exudation. (B) Bright field image of immature (lower) radicle, exhibiting no visible root exudation. (C) Fluorescent image of (A) with Texas red filter showing corresponding shikonin localization in same tissue sample. (D) Fluorescent image of (B) with Texas red filter showing absence of shikonin autofluorescence. Bars, 200 µm (A–D).

Time course experiments performed in this study at short intervals following seed imbibition also yielded important information regarding phenological development of seedling root hairs and periderm tissues over time (Fig. 3A–E). Deposition of coloured shikonins was first observed in *E. plantagineum* seedlings at ~48h following imbibition of seed and consistently appeared in the root–hypocotyl junction. In addition, nearby numerous root hairs on the developing radicle were observed exuding copious quantities of bright red exudates as droplets at the tip of the root hair (Fig. 3C, F). When contrasting the production of shikonins in various locations along a single seedling root, we noted that the basal portion of the seedling root possessed a higher level of active root hairs and greater numbers of shikonin-producing periderm cells than did the acropetal portion of the same root at 72h following imbibition (Fig. 4A–D). With the presence of significant quantities of shikonin in more mature periderm tissue and root hairs, we noted distinct autofluorescence due to the presence of these compounds in the basal portion of the root and not in the acropetal. The bright red autofluoresence observed in Fig. 4C was associated with the exudation of root hairs containing concentrated levels of shikonins.

### Screening of Australian populations of E. plantagineum

**Fig. 5.**
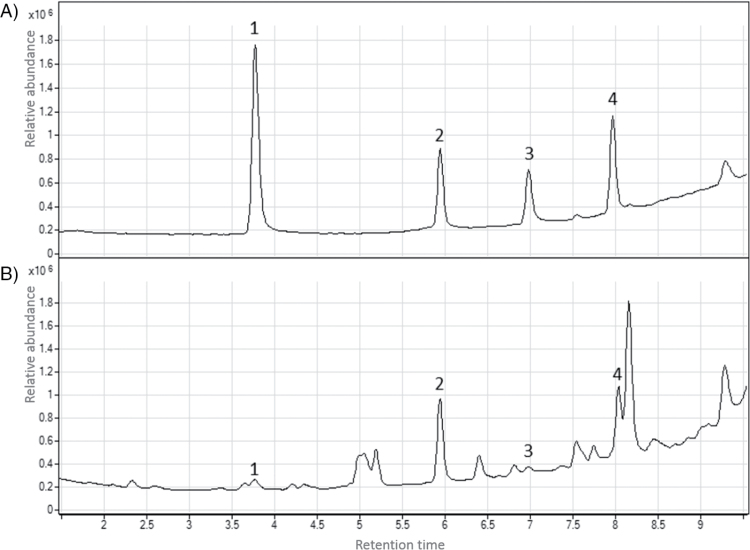
Total ion chromatograms obtained using UHPLC/Q-ToF MS for a prepared mixture of analytical standards (A) in comparison to a standard periderm extract (B). Identified compounds include: 1, shikonin; 2, acetylshikonin; 3, deoxyshikonin; 4, dimethylacrylshikonin.

UHPLC/Q-ToF MS separation and detection resulted in successful metabolic profiling of numerous related and bioactive shikonins in field-grown plants; specifically, we detected and identified shikonins at significant and potentially bioactive as phytotoxins and antimicrobials ranging from 0.3 to 10ppm in periderm extracts resulting from direct ethanolic extraction from plants of 21 geographically distinct populations (Garcia Duran *et al.*, 2014, 2015). Concentrations of shikonins present in intact periderm are estimated to be potentially higher in some periderm tissues, based on confocal experimentation and hyperspectral imaging studies, and estimation in intact tissues is dependent on rooting environment and plant maturity. Although Weston *et al.* (2013b) previously reported the presence of acetylshikonin, deoxyshikonin and shikonin in root extracts using an Agilent LC/MS 6410 QQQ instrument, in this experiment using an Agilent 6530 UHPLC/Q-ToF MS, the presence of numerous additional (>9) shikonin derivatives in samples obtained from plants collected across southern Australia was more precisely noted using similar methods to those reported in Skoneczny *et al.* (2014, 2016). By direct comparison with available purified standards of four derivatives, the abundance of three of the most bioactive NQs in periderm extracts including acetylshikonin, deoxyshikonin and shikonin (Garcia Duran *et al.*, 2014) as well as dimethylacrylshikonin in all extracts was also assessed (Fig. 5A, B).

**Fig. 6.**
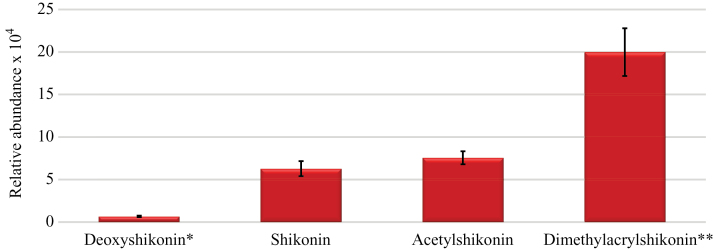
Relative abundance of shikonins in periderm extracts collected from geographically distinct populations of *E. plantagineum* across NSW, Australia in 2012–2014. Samples were analysed using UHPLC/Q-ToF MS instrumentation using the tandem mass spectrometry approach in comparison to purified standards of the same NQ derivatives. Each sample was a composite of 5–6 individual collected from 21 populations and results were averaged for each compound of interest. Error bars denote standard error of the means, *n*=21. *, compound in significantly lower abundance (*P*<0.001); **, compound in significantly higher abundance (*P*<0.001).

Despite variation among samples, we found a consistent pattern of compound abundance among root periderm extracts from mature field-collected plant populations (Fig. 6). Dimethylacrylshikonin was present in significantly higher abundance (*P*<0.001) in all field periderm extracts, while deoxyshikonin was present in significantly lower abundance (*P*<0.001) in all samples. Deoxyshikonin is thought to be the precursor of shikonin and is therefore likely rapidly converted to shikonin and also potentially numerous higher molecular weight derivatives, particularly dimethylacrylshikonin (Papageorgiou *et al.*, 1999; Sommer *et al.*, 1999). Shikonin, acetylshikonin and dimethylacrylshikonin were all highly active when assessed as either plant growth inhibitors or antimicrobials, with shikonin generally the most active (Weston *et al.*, 2012b; Garcia Duran *et al.*, 2014). We have identified other NQ derivatives in root extracts of various *Echium* spp. in trace quantities, but their biological activity at this time is not known. Only one population (White Cliffs, NSW, Australia) showed greatly enhanced production of shikonins, specifically dimethylacrylshikonin, in contrast to the other 20 populations evaluated. Interestingly, plants in White Cliffs are typically exposed to high UV and summer temperatures and low average rainfall events, as this location borders the Australian outback, a large inland desert. The evaluation of gene expression underlying biosynthesis of dimethylacrylshikonin and other NQs in this population is now underway.

### Analysis of soil and microbe extracts

**Fig. 7.**
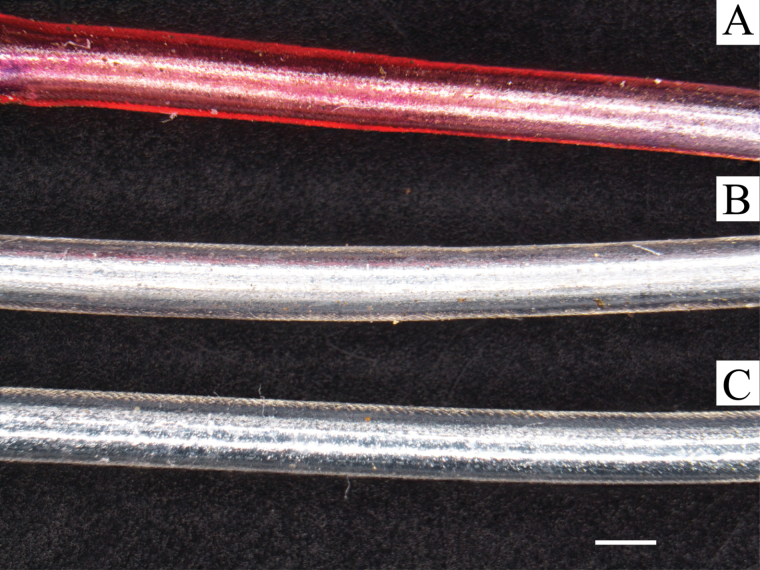
SPRE microprobes consisting of PDMS tubing placed over stainless steel 22 gauge wire (Weidenhamer *et al.*, 2009). Images A–C represent varying concentrations of NQs typically extracted by microprobes from the rhizosphere of *E. plantagineum* grown in pot experiments in the glasshouse. Bar, 1mm. Image C also appears visually identical to control probes and/or those not significantly exposed to root-infested soil.

Using an HPLC/MS Q-Trap for sensitive analysis of NQs in extracts and soils, we developed a reliable multiple reaction mode (MRM) method that allowed detection of trace levels of selected shikonins in both soil and microprobe extracts. The main constituent found in microprobes brought into contact manually with living *E. plantagineum* roots or placed in soil of glasshouse-grown potted plants was acetylshikonin, observed in probe extracts at levels of ~0.9ppm and in ethanolic extracts of soil at levels of 2ppm using ethanolic extraction. Our limit of detection (LOD) of acetylshikonin in soil and microprobe samples subjected to potted soils and roots was ~0.3ppm. In all probe and soil extracts collected from potted soil rhizospheres surrounding mature *E. plantagineum*, shikonin was the next most abundant compound (Table 1). Deoxyshikonin, dimethylshikonin and other shikonin derivatives were often below the limit of detection (LOD) in most samples (Table 2). Interestingly, shikonin and acetylshikonin are typically the most phytotoxic of the shikonin type of NQs studied (Garcia Duran *et al.*, 2014, 2015), and acetylshikonin particularly appears to accumulate in highest concentrations in soil and periderm extracts over time. This may be associated with the relative stability of shikonin and acetylshikonin, in contrast to their precursor deoxyshikonin or larger molecular weight derivatives. However, these studies clearly show that NQs do accumulate in significant levels in the rhizosphere of living plants. Not only can shikonins be directly extracted from soil, small soil microprobes can be utilized to more accurately detect their presence within the living root system. Microprobes have been previously successfully employed for direct solid phase extraction of non-polar constituents such as the thiophenes or sorgoleones from soil (Weidenhamer *et al.*, 2009), and in this case the technique worked well to extract considerable quantities of moderately non-polar shikonin derivatives from soil, as reported by Weidenhamer *et al.* (2014). Entrapment of shikonins on microprobes was easily observed by the red or pink colour of microprobes placed in contact with living roots or removed from the rhizosphere (Fig. 7) of living potted plants.

**Table 2.** Presence of four bioactive shikonins identified by tandem MS and studied in different matrices in microprobe experimentation LOQ, limit of quantification; LOD, limit of detection.

Type of extract	Acetylshikonin	Deoxyshikonin	Dimethylscrylshikonin	Shikonin
Periderm	>LOQ	>LOQ	>LOQ	>LOQ
Silicone tubing	>LOQ	<LOD	<LOD	<LOD
Soil and silicone tubing	>LOQ	<LOD	<LOD	=LOD

The role of various root-produced naphthoquinones such as shikonins and their derivatives has been little described in the literature of higher plants under natural field settings, particularly those that are classified as invasive weeds of significance. Interestingly, we have found that *E. plantagineum* plants produced in several climatic zones in Spain contained >2-fold lower concentrations of shikonins than plants produced in field conditions across southern Australia (unpublished data; Garcia Duran, 2014, 2015). These findings are similar to those of Thorpe *et al.* (2009), who detected greater bioactivity of *Centaurea maculosa* root exudates and bioactive PSPs on plants collected in invasive versus native ranges. We are currently further examining the role of environment and genetics upon regulation of NQ production in *Echium* spp. However, our findings here, in combination with our field and controlled environment experiments (Garcia Duran *et al.*, 2014; Skoneczny *et al.*, in press) suggest that shikonins play an important role in plant protection against microbial invaders, insect herbivores and germinating plants in the rhizosphere of *E. plantagineum*. These studies utilized a combination of intricate approaches to study the localization and distribution of bioactive shikonins in the plant and rhizosphere directly surrounding living *E. plantagineum*. The reported biological activity and observed accumulation of shikonins and other NQ derivatives in the periderm and their subsequent deposition in the rhizosphere through exudation or tissue degradation are all suggestive of their potential importance as bioactive novel weapons of significance. This is likely to be of particular importance in monocultural stands under warm and dry conditions in Australia where shikonin production was noted to be enhanced. Studies are now underway to further evaluate and compare NQ and pyrrolizidine alkaloid production and genetics of *E. plantagineum* and other related species in Australia and their native range in the Iberian Peninsula.
